# The Microstructure, Solidification Path, and Microhardness of As-Cast Ni-Al-Cr-Os Alloys in a Ni-Rich Region

**DOI:** 10.3390/ma16206777

**Published:** 2023-10-20

**Authors:** Yan Lin, Ming Wei, Guangyu Yang, Haiyan Liu, Hui Ye, Chunming Deng, Lijun Zhang

**Affiliations:** 1 State Key Laboratory of Porous Metal Materials, Northwest Institute for Non-Ferrous Metal Research, Xi’an 710016, China; mingwei_mse@sina.com (M.W.); yanggy0403@163.com (G.Y.); liuhaiyan1108@163.com (H.L.); ninhuiye@126.com (H.Y.); 2 Institute of New Materials, Guangdong Academy of Sciences, National Engineering Laboratory for Modern Materials Surface Engineering Technology, The Key Lab of Guangdong for Modern Surface Engineering Technology, Guangzhou 510651, China; dengchunming@gdinm.com; 3 State Key Laboratory of Powder Metallurgy, Central South University, Changsha 410083, China

**Keywords:** Ni-based superalloy, Ni-Al-Cr-Os, as-cast microstructure, solidification path, microhardness

## Abstract

In this study, nine as-cast Ni-Al-Cr-Os alloys were prepared, and their constituent phases and microstructure were examined using X-ray diffraction and electron probe microanalysis techniques. The solidification paths of all the alloys in a Ni-rich corner were revealed based on a detailed analysis of the as-cast microstructure. The liquidus cube of the quaternary Ni-Al-Cr-Os system in a Ni-rich corner was established accordingly. A eutectic-type invariant reaction on the liquidus surface was explicitly identified, and its reaction can be expressed as L → α + β + γ. No quaternary invariant reaction was found in the alloys following the addition of Os. The Ni-Al-Cr-Os alloy points were then vertically mapped onto the Ni-Al-Cr liquid phase projection to better observe the effect of Os addition on the solidification path of the Ni-Al-Cr system. It was found that the addition of a small amount of Os has no significant effect on the solidification path of the Ni-Al-Cr system. Furthermore, the microhardness of each alloy, which was determined to be in the range of 207 HV to 565 HV, was found to be closely related to the phase constitution and phase fraction of the alloy.

## 1. Introduction

Nickel-based single-crystal superalloys are widely applied as high-temperature-resistant materials in the aerospace domain on account of their superior mechanical properties [1,2,3,4]. In order to fulfill the increasing requirements for better performance under extreme conditions, the manufacturing of novel nickel-based single-crystal superalloys with properties suitable for high-temperature, mechanical, and creep resistance needs to be investigated, e.g., by adding different alloying elements [5,6,7]. One research hot spot in this field lies in the development of new single-crystal superalloys with less or even no Re [8,9,10,11]. In our latest studies on the determination of interdiffusion coefficients in a series of nickel binary and ternary alloys [12,13], our research group discovered that the interdiffusion coefficients in nickel-based alloys containing Os element are lower than those containing Re when the temperature is above 1300 °C. Therefore, we conclude that the addition of Os to nickel-based single-crystal superalloys may help to retain or even improve their creep resistance.

However, whether Os can be an alternative element for replacement of element Re in new-generation nickel-based, single-crystal superalloys requires further consideration of more factors. Accurate phase equilibria and/or thermodynamic descriptions of nickel-based alloys with Os are both essential; unfortunately, relevant research on such topics remains rare [14,15]. In order to remedy this situation, a project that measured the accurate phase equilibria in nickel-based alloys with Os and established their accurate thermodynamic database was carried out by our research group [12,16]. In 2015, Chen et al. [12] performed a thermodynamic assessment of a binary Ni-Os system based on the experimental phase equilibria reported in the literature [15]. After that, three of the present authors [16] conducted a thorough experimental measurement of the isothermal sections of a Ni-Al-Os system at 1000 and 1200 °C, as well as the isothermal tetrahedron of a Ni-Al-Cr-Os system at 1200 °C in a Ni-rich region. Based on the current phase equilibria in nickel-based alloys with Os, a discussion on the possible substitution of Re with Os in new-generation nickel-based single-crystal superalloys was conducted, resulting in some promising but still tentative conclusions [16]. After that, based on experimental information and valuable assertions [13,16], Wei et al. [17] attempted to develop new nickel-based single-crystal superalloys by replacing Re in commercial CMSX-4 with Os. It was observed that this new superalloy had better microstructural stability and creep resistance than CMSX-4. Thus, it is certain that the replacement of Re by Os in nickel-based single-crystal superalloys is feasible. However, in order to accurately design optimal alloy compositions and processing mechanisms for nickel-based single-crystal superalloys containing Os, a continuous study of the fundamental properties of different Ni alloys with Os is necessary.

In nickel-based single-crystal superalloys, solidification characteristics play an important role in the evolution of as-cast structures [18], the precipitation and distribution of the secondary phases, and the formation of casting defects. In particular, the addition of a large number of refractory elements [19], such as Ta, Mo, W, Re, etc., leads to more serious microsegregation during the solidification process. Thus, the tendency of grain defects occurring and the precipitation probability of a harmful topological close-packing (TCP) phase [20] are increased. This makes subsequent heat treatment more difficult and ultimately affects the mechanical properties of the alloy. By studying a correlation phase diagram [21,22], we learned that Os and some other refractory elements are likely to produce TCP phases. Therefore, it is valuable to study the solidification characteristics of relevant as-cast nickel-based superalloys that contain Os and to optimize their composition and treatment to obtain the desired properties, further avoiding the formation of potentially harmful topological close-packed phases.

Despite the composition of commercial nickel-based superalloys, the Ni-Al-Cr system provides the basis for most Ni-based superalloys. Consequently, the Ni-Al-Cr-Os quaternary system is studied in this paper. The major research objectives are as follows: (i) to measure the microstructure and microhardness of as-cast Ni-Al-Cr-Os alloys in a Ni-rich region and (ii) to analyze their solidification paths and establish the relation between microhardness and as-cast structures.

## 2. Experimental Procedure

In this study, nine quaternary Ni-Al-Cr-Os samples with around 5 wt.% Os were selected. All the samples were smelted by melting a mixture of raw materials—Ni, Al, Cr, and Os (purity: >99.9%, provided by China New Metal Materials Technology Co., Ltd., Beijing, China)—in an arc melting furnace (WK-I, Physcience Opto-electronics Co., Ltd., Beijing, China) under a high-purity argon atmosphere in a water-cooled copper hearth with a non-consumable tungsten electrode, with titanium metal pieces used as getters. In order to ensure that the refractory element (Os) was adequately and uniformly melted into the alloy ingot, samples were prepared according to the following three steps [12]. The master Ni-Os ingots were melted first. Then, the Ni-Os ingots were cut into small pieces and remelted a few times to ensure that the Os had adequately melted into the alloy ingots. The appropriate Al + Cr ingots were added to the premelted Ni-Os ingots and melted in the last step. During the process, each ingot was smelted five times for homogenization. The mass loss for each step was maintained at less than 1 wt.%. The actual alloy compositions for the Ni-Al-Cr-Os alloys are summarized in Table 1.

The samples used for microstructural analysis were polished using standard metallographic techniques. Microstructural investigations and the determination of phase compositions were carried out for all samples using the electron probe microanalysis (EPMA) (JXA-8100, JEOL, Tokyo, Japan) technique. The microstructural features were quantitatively acquired using image analysis software (Image J 1.8.0). The X-ray diffraction (XRD) patterns were examined using an X-ray diffractometer (D8 Advance, Bruker, Karlsruhe, Germany) with Cu-Ka radiation at 40 kV and 40 mA. Data in a 2θ range of 20° to 90° were collected. The microhardness of all nine samples was measured using a microhardness tester (HVS-1000A, HUAYIN Ltd., Laizhou, China) equipped with a Vickers diamond indenter. A load of 9.8 N was applied for 15 s. Five measurements were carried out for each sample.

## 3. Results and Discussion

### 3.1. Microstructures of As-Cast Ni-Al-Cr-Os Quaternary Alloys

**Figure 1:**
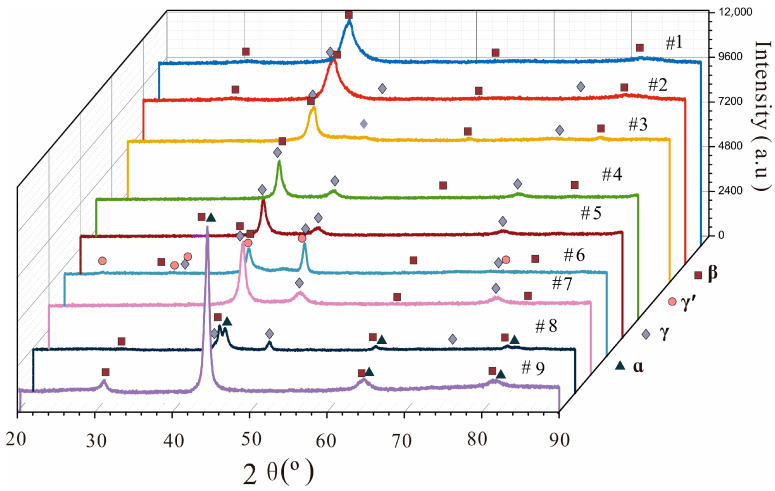
(Color on line) XRD patterns of all nine as-cast Ni-Al-Cr-Os alloys investigated in the present study.

**Figure 2:**
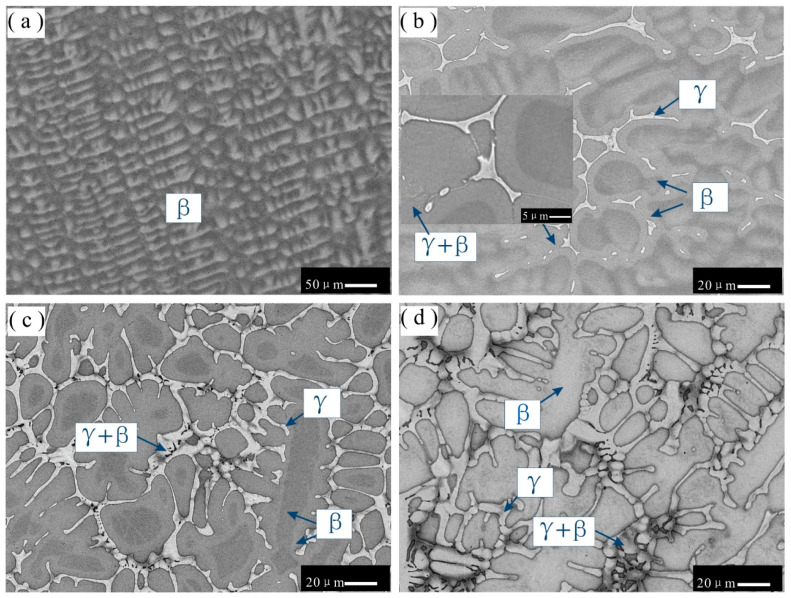
BSE images of as-cast microstructures in different alloys: (**a**) alloy 1; (**b**) alloy 2; (**c**) alloy 3; (**d**) alloy 7. Single-phase β dendritic morphology in alloy 1. Gray primary phase β in alloys 2, 3, and 7 and pale gray phase γ formed between the interdendritic β phase.

**Figure 3:**
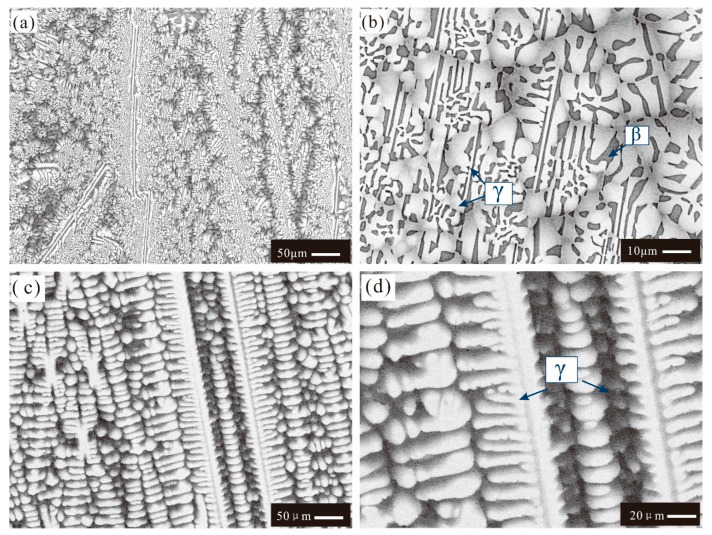
BSE images of the as-cast microstructure in different alloys: (**a**,**b**) alloy 4; (**c**,**d**) alloy 5. White strip phase primary γ phase surrounded by a cluster of eutectic morphology (i.e., white phase γ plus gray phase β) in alloy 4. γ single-phase dendritic morphology in alloy 5.

**Figure 4:**
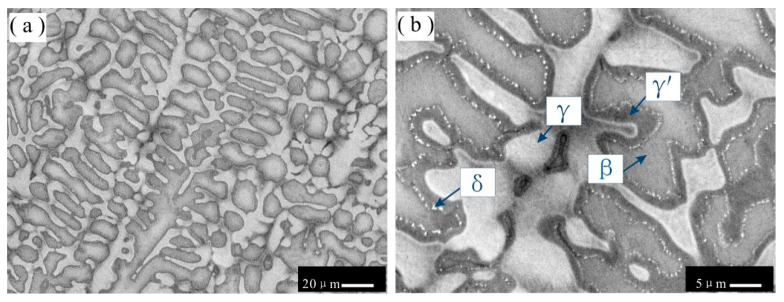
BSE images of as-cast microstructures in alloy 6 at (**a**) low magnification and (**b**) high magnification. Gray primary phase (β) surrounded by dark gray phase (γʹ) in alloy 4; the light gray phase (γ) occurs in the interdendritic space, and fine white particles form at the edge of the dark gray γʹ phase.

**Figure 5:**
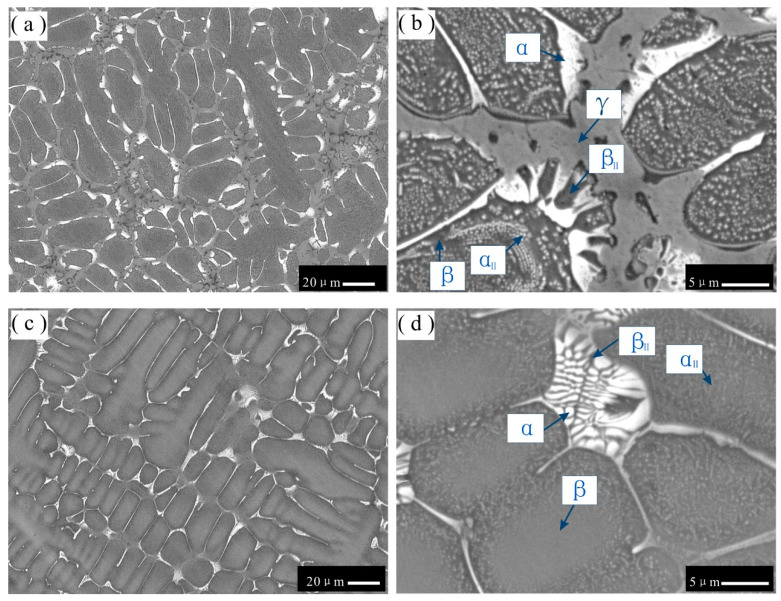
BSE images of as-cast microstructures in different alloys: (**a**,**b**) alloy 8; (**c**,**d**) alloy 9. Three-phase eutectic structure (i.e., white phase (α), gray phase (γ), and dark gray phase (β)) formed in the interdendritic space of primary phase β in alloy 8. Two-phase eutectic structure (i.e., white phase (α) and gray phase (β)) formed in the interdendritic space of the primary phase (β) in alloy 9. A large number of fine white particles precipitated in the primary phase (β) in alloys 8,9.

The phases and their fractions in the nine as-cast Ni-Al-Cr-Os alloys, as determined using XRD and EPMA analyses, are listed in Table 1. For crystallographic information on the γ, γ’, δ, β, and α phases, readers should refer to Table 2 in our previous publication [16]. The XRD patterns for all as-cast Ni-Al-Cr-Os alloys are presented in Figure 1, while as-cast microstructures are displayed in Figure 2, Figure 3, Figure 4 and Figure 5. 

As indicated in Figure 1, the XRD patterns show that the phases of the samples are mainly β or γ phases. α and γʹ phases are present in only a few samples. Among these, alloys 1 and 5 are single β and γ phases, respectively; alloys 2, 3, 4, and 7 are all two-phase β and γ phases; alloy 9 comprises two-phase β and α phases; alloy 8 contains three-phase β, γ, and α phases; and alloy 6 mainly comprises three-phase β, γ, and γʹ phases.

Figure 2a presents a backscattered electron (BSE) micrograph of as-cast alloy 1, in which only single-phase dendrites are present. As corroborated by the XRD results, alloy 1 is confirmed to be in the single-phase region of β. Moreover, as shown in Figure 2a, the central part of the β dendrites is light gray, while the interdendritic region appears as dark gray. The EPMA results show that the content of Os in the central part of β dendrites is 6.5 wt.%, while that in the edge of β dendrites is only 2.3 wt.%. This microsegregation phenomenon may be due to the low diffusion rate of Os, which does not allow for sufficient diffusion; thus, a homogenous distribution cannot be achieved during the solidification process.

Figure 2b–d show BSE micrographs of as-cast alloys 2, 3, and 7, respectively. As shown in the figures, the microstructures of alloys 2, 3, and 7 comprise a dendritic morphology and a eutectic-like morphology across the interdendritic region. Based on the XRD and EMPA results, the gray phase is confirmed as the β phase, while the pale gray phase is the γ phase. Similarly, in Figure 2b,c, considering the noticeable contrast in the central (dark gray) and edge (gray) regions of β dendrites, a microsegregation phenomenon is observed in the β dendrites of alloys 2 and 3. According to the point measurement conducted using the EPMA technique, the compositions of Al and Os in the central region of β dendrites of both alloys 2 and 3 are larger than those in the edge region by 2.7 and 1.5 wt.%, respectively. The Cr composition in the central region of β dendrites of both alloys 2 and 3 is lower than that in the edge region by 3.6 wt.%. The central (dark gray) region of the β phase in the BSE micrograph may be caused by the inclusion of a slightly higher amount of Al with a low atomic number.

Figure 3 displays BSE images of as-cast alloys 4 and 5. As shown in Figure 3a,b, the as-cast microstructure of alloy 4 has a eutectic morphology (i.e., white phase plus gray phase) and some white strip phases over the eutectic region. The XRD and EPMA results confirm that the gray phase is β, while the white/white strip phase is γ. It can be clearly seen in Figure 3c,d that the as-cast microstructure of alloy 5 shows a typical single-phase (i.e., a γ phase according to the XRD results) dendritic morphology. Moreover, a noticeable color contrast between the central region (light gray) and the edge region (dark gray) of the γ dendrites is observed in Figure 3c,d, indicating the noticeable microsegregation phenomenon in γ dendrites during solidification. The point composition measurement conducted using EPMA shows that the content of Os in the central region of the γ dendrites is 6.3 wt.%, while that in the edge region is only 1.3 wt.%.

BSE images of the as-cast microstructures in alloy 6 are shown in Figure 4a,b at different magnifications. The higher-magnification BSE image shown in Figure 4b shows that as-cast alloy 6 has a complex microstructure with four phases. The gray phase is surrounded by a dark gray phase. The remaining wide region of the as-cast microstructure is occupied by the light gray phase. The XRD and EPMA results confirm that the gray phase, the dark gray phase, and the light gray phase are β, γʹ, and γ, respectively. Moreover, some fine white particles, which may form via solid-state transformation, are observed along the grain boundaries of the dark gray phase (γʹ) and are regarded as the δ phase.

The as-cast microstructure of alloy 8 is exhibited in Figure 5a,b. Figure 5a illustrates a dendritic morphology, which is identified as the β phase according to XRD and EPMA characterizations. Aside from the β dendrites, the higher-magnification image of the as-cast microstructure in Figure 5b has a eutectic morphology consisting of one gray phase, one dark gray phase, and one white phase over the interdendritic region. Combined with the XRD and EPMA results, the gray phase was determined to be γ, the dark gray phase was determined as β, and the white phase was determined as α. There are also some very fine white particles (i.e., α phase), which may be also induced by the solid-state transformation, appearing in the β phase.

The morphology of the as-cast microstructures in alloy 9 is displayed in Figure 5c,d with different magnifications. In Figure 5c, the dendritic β phase can be observed, which is similar to the as-cast microstructure of alloy 8, as shown in Figure 5a. However, the magnified BSE image in Figure 5d has a different microstructure compared to that of alloy 8. According to Figure 5d, the eutectic structure of as-cast alloy 9 consists of only two phases (i.e., one gray phase plus one white phase). The white phase is identified as α, while the gray phase is β. Similarly, one can also find some very fine white α particles, which are formed via solid-state transformation in the β phase.

### 3.2. Solidification Path of Ni-Al-Cr-Os Quaternary Alloys

Based on the above results and analysis of as-cast microstructures, alloy 1 only consists of a single β phase, indicating that the β phase is directly solidified from the liquid phase.

In as-cast alloys 2, 3, and 7, the primary phase is β, and a coarse eutectic reaction occurs, resulting in the formation of the γ phase. Thus, the solidification sequence of alloys 2, 3, and 7 can be proposed as follows:

L→β

L→β+γ

The BSE image of alloy 4 shows that the alloy consists of a large volume fraction of eutectic morphology (γ + β) and a low volume fraction of a white strip of γ phase in the eutectic structure. This demonstrates that the composition of alloy 4 should be relatively close to the eutectic point of L→ β + γ. The as-cast microstructure shows that the primary phase may be combined the white strip γ phase; then, the eutectic structure is formed by the remaining liquid phase. Therefore, the solidification path of alloy 4 is proposed as follows:

L→γ

L→β +γ

As-cast alloy 5 has a simple dendritic structure, which indicates that the γ phase is completely formed directly from the liquid. 

In as-cast alloy 6, the dendritic β phase is the primary phase during the solidification process. The significantly irregular outline of the β boundaries indicates that it is associated with a peritectic or divorced eutectic reaction, leading to the formation of the γʹ phase. Then, the γ phase is formed directly from the remaining liquid phase due to its relatively fast cooling rate. Therefore, the solidification sequence of alloy 6 is proposed as follows:

L→β

$$L+\beta \to \gamma^{\prime }\;\mathrm{or}\;L\to \gamma^{\prime }+\beta$$

L→γ

In as-cast alloy 8, the primary phase should be the β dendrites. Moreover, since the eutectic structure of β + γ + α is located in the interdendritic region, a eutectic reaction may directly occur from the remaining liquid phase after the formation of the primary β phase. The solidification path of alloy 8 is proposed as follows:

L→β

L→β+γ +α

In as-cast alloy 9, the eutectic structure of (β + α) is distributed over the interdendritic region of primary β dendrites. On account of the as-cast microstructure, the solidification path of alloy 9 is proposed as follows:

L→β

L→β +α

**Figure 6:**
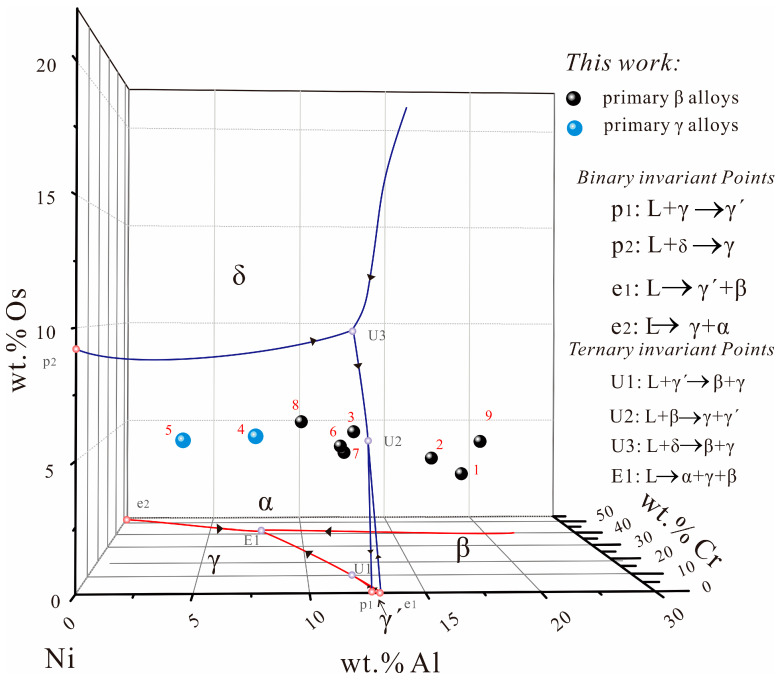
(Color on line) Liquidus cube of the Ni-Al-Cr-Os quaternary system in the constructed Ni-rich region.

By combining the above experimental results with the phase equilibria in three boundary binary systems (i.e., Ni-Al [23], Ni-Cr [24,25], and Ni-Os [15]) and two boundary ternary systems (i.e., Ni-Al-Cr [23,24,25,26] and Ni-Al-Os [16]), the liquidus projection of the Ni-Al-Cr-Os quaternary system in a Ni-rich corner is established in Figure 6. The selected alloys and their primary phases are indicated in this plot. As shown in Figure 6, five primary solidification surfaces—the β, γ, γ′, δ, and α phases—appear in the liquidus projection. The primary phases of alloys 4 and 5 are γ phases, and those of the remaining alloys are β phases. Moreover, a eutectic-type invariant reaction (E1) on the liquidus surface can clearly be observed; this reaction yields L → α + β + γ. Furthermore, in this study, no quaternary invariant reaction was observed following the addition of Os.

**Figure 7:**
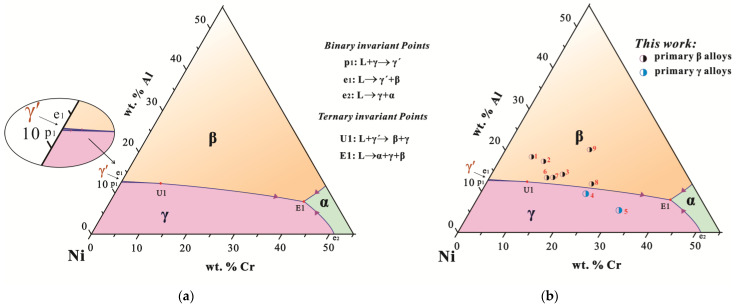
(Color on line) Comparison between liquidus projections of (**a**) the ternary Ni-Al-Cr system in a Ni-rich region from [23,24,25,26] and (**b**) the quaternary Ni-Al-Cr-Os system with around 5 wt.% Os in a Ni-rich region.

In order to better observe the effect of Os addition on the solidification path of the Ni-Al-Cr system, the Ni-Al-Cr-Os alloy points were vertically mapped to the Ni-Al-Cr liquid phase projection diagram, as shown in Figure 7. As displayed in Figure 7a, there are four primary solidification surfaces in the Ni-Al-Cr liquidus projection—the β, γ, γ′, and α phases. As shown in Figure 7b, alloys 4 and 5 fall on the γ phase solidification surface, while the remaining alloys fall on the β phase solidification surface. The primary phase region of the Ni-Al-Cr-Os alloy points in the Ni-Al-Cr liquid projection diagram is consistent with the primary phase results of the Ni-Al-Cr-Os alloys obtained in the experiments. It is concluded that the addition of around 5 wt.% Os has no significant effect on the solidification path of the Ni-Al-Cr system.

### 3.3. Microhardness Values of As-Cast Ni-Al-Cr-Os Quaternary Alloys and Their Correlation with As-Cast Microstructures

**Figure 8:**
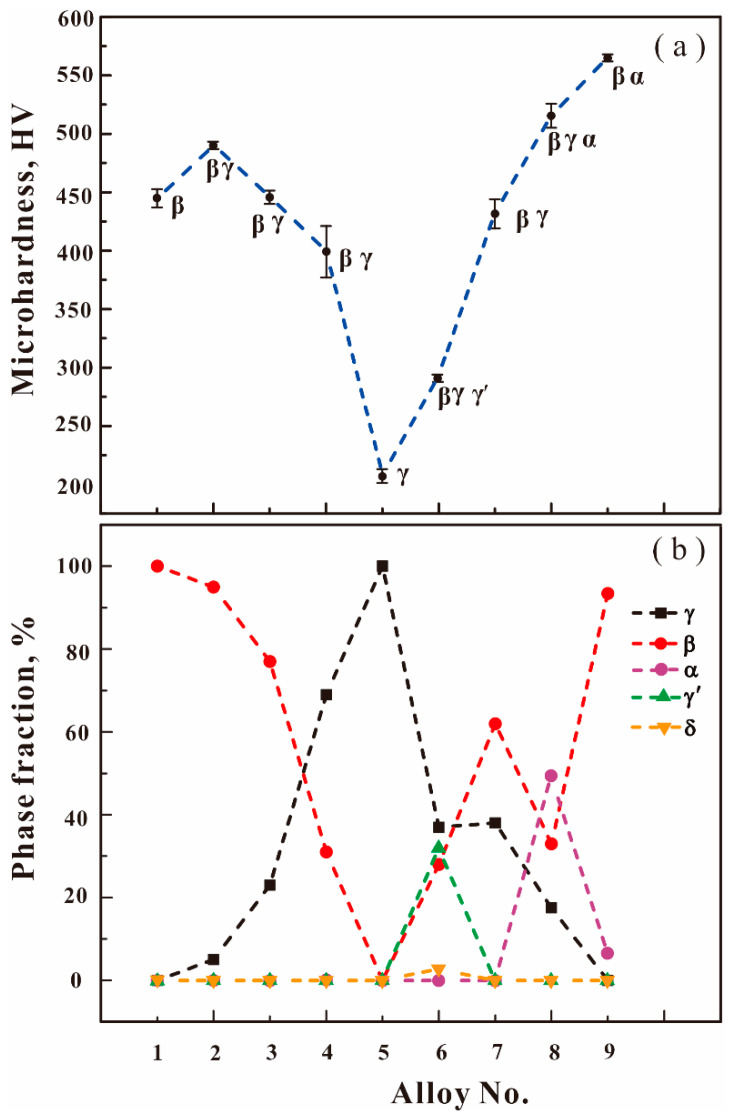
(Color on line) Measured (**a**) microhardness values and (**b**) fractions of the constituent phases in nine as-cast Ni-Al-Cr-Os alloys.

Figure 8a shows the evolution of the measured microhardness of all nine as-cast Ni-Al-Cr-Os quaternary alloys. It can be seen in Figure 8a that the microhardness of all the as-cast alloys varies between 207 HV and 565 HV. The constituent phases in each as-cast alloy are also shown in Figure 8a. In order to understand the effect of as-cast microstructure on microhardness, the fractions of different constituent phases in each as-cast alloy are displayed in Figure 8b. By comparing Figure 8a,b, one can analyze the correlation of microhardness with the as-cast microstructures in the as-cast Ni-Al-Cr-Os quaternary alloys, as discussed in detail below.

Different constituent phases with different crystal structures and fractions make different contributions to the microhardness. Two minima of microhardness (i.e., 445 HV and 207 HV) precisely correspond to alloys 1 and 5, respectively. This is because only a single phase exists in both alloys 1 (single β phase) and 5 (single γ phase). It can also be inferred that the β phase is much harder than the γ phase. Thus, from alloy 2 to alloy 5, the alloy hardness clearly decreases with the decrease in the β phase fraction and the increase in the γ phase fraction. Furthermore, a small amount of the γ′, δ, and/or α phases is found in alloys 6 to 9, which also causes a change in microhardness.

Through the aforementioned research, a comprehensive investigation of the as-cast microstructural characteristics, solidification path, and microhardness of a nickel-rich Ni-Al-Cr alloy containing 5 wt.% Os was conducted. The solidification characteristics of the superalloys were thoroughly examined, leading to the development of a liquid phase projection for the Ni-Al-Cr-Os system in a Ni-rich corner. Subsequent analysis revealed that during the solidification process of nickel-based superalloys containing about 5 wt.% Os, γ, β γ′, δ and/or α phases are present in the solidification structure, while no TCP phase is formed. The calculated isopleth of the Ni-Al-Cr-Re system [27] with 1.5 at.% Re, X_Ni_/X_Al_ = 1, indicates the existence of some phase regions associated with a σ-TCP phase. It was observed that during the solidification process, the addition of around 5 wt.% Os did not significantly alter the solidification path of the Ni-Al-Cr system in the Ni-rich end. In the as-cast Ni-Al-Cr-Os alloys, the β phase predominantly contributes to the hardness of alloys, while the γ phase acts as a softer constituent. The relative fraction of these phases directly influences the overall hardness value of the alloy. 

This research further expands knowledge of the solidification characteristics and mechanical properties of nickel-based superalloys containing Os, providing experimental support for the establishment of thermodynamic data so as to further accelerate the design of a new generation of nickel-based single-crystal superalloys containing Os.

## 4. Summary

In order to investigate the solidification characteristics of nickel-based superalloys containing Os, nine quaternary Ni-Al-Cr-Os samples with around 5 wt.% Os were prepared using an arc melting technique. Based on XRD and EPMA analyses, the constituent phases and microstructures in all as-cast Ni-Al-Cr-Os alloys were examined. The main conclusions of this study are reported as follows. The solidification path for each alloy was determined based on a comprehensive analysis of as-cast microstructures. The liquidus cube for the Ni-Al-Cr-Os quaternary system in a Ni-rich corner was established. The liquidus cube consists of five primary solidification regions: the β, γ, γ′, δ, and α phases. A eutectic-type invariant reaction (E1) on the liquidus surface was effectively conducted, which is expressed as L → α + β + γ. The Ni-Al-Cr-Os alloy points were vertically mapped to the Ni-Al-Cr liquid phase projection diagram. The primary phase region of Ni-Al-Cr-Os alloys was consistent with experimental results. It is concluded that the addition of a small amount of Os did not significantly affect the solidification path of the Ni-Al-Cr system. The microhardness values of all as-cast alloys were measured, and their correlations with microstructure were also studied. It was observed that the hardness of alloys changes from 293 HV to 565 HV and is highly dependent on the constituent phases and phase fractions of the as-cast alloys. The β phase is much harder than the γ phase. Thus, from alloy 2 to alloy 5, hardness decreases with a decrease in the β phase fraction and the increase in the γ phase fraction. Furthermore, a small amount of the γ′, δ, and/or α phases is found in alloys 6 to 9, which also causes a change in microhardness.

In this study, we determined the solidification characteristics of nickel-based alloys containing Os. However, further research on this topic is necessary. In order to effectively design nickel-based single-crystal superalloys containing Os with exceptional properties, it is essential to investigate both as-cast and equilibrium Ni-Al-Cr-Os-M alloys (where M represents all other key components of Ni-based superalloys) and obtain a comprehensive multicomponent phase diagram. This will provide essential thermodynamic data for the design of nickel-based single-crystal superalloys containing Os.

## Figures and Tables

**Table 1:** List of the actual compositions of as-cast alloys, as well as the phases, phase fractions, and solidification paths in their as-cast microstructures based on the present phase/microstructural analyses (XRD and EPMA techniques).

No.	Alloy Composition (wt.%)	Phase	Solidification Path	Phase Fraction
1	Ni_74.0_Al_16.8_Cr_4.9_Os_4.3_	β	L → β	100% β
2	Ni_71.2_Al_15.6_Cr_7.8_Os_4.8_	β, γ	L → β L → β + γ	95% β, 5% γ
3	Ni_69.1_Al_12.2_Cr_13.0_Os_5.7_	β, γ	L → β L → β + γ	77% β, 23% γ
4	Ni_66.7_Al_7.6_Cr_20.4_Os_5.3_	γ, β	L → γ L → γ + β	31% β, 69% γ
5	Ni_61.9_Al_3.8_Cr_29.5_Os_4.8_	γ	L → γ	100% γ
6	Ni_73.0_Al_11.5_Cr_10.3_Os_5.2_	β, γ, γʹ, δ	L → β L + β → γʹ or L→ β + γʹ L →γ	28.2% β, 37% γ, 32% γʹ, 2.8% δ
7	Ni_71.9_Al_11.7_Cr_11.5_Os_4.9_	β, γ	L → β L → β + γ	62% β, 38% γ
8	Ni_64.0_Al_9.8_Cr_20.2_Os_5.9_	β, γ, α	L → β L → β +γ + α	33% β, 17.5% γ, 49.5% α
9	Ni_60.6_Al_18.3_Cr_15.9_Os_5.2_	β, α	L → β L → β + α	93.4% β, 6.6% α

## Data Availability

The data supporting the findings of this study are available within the article.
